# Phenotypic and Molecular Assessment of *Candida* Species and Real-Time PCR Detection of Selected Virulence Genes in Immunocompromised Patients Admitted to Intensive Care Units

**DOI:** 10.3390/pathogens15080878

**Published:** 2026-08-21

**Authors:** Hala Altarawneh, Eman H. M. Salem, Asmaa M. Soliman, Amany T. Elfakhrany, Heba M. Abdelkareem, Yasmin Mohsen, Abdallah M. Gameel, Hala A. E. Khalil, Rasha G. Mostafa

**Affiliations:** 1 Department of Microbiology and Pathology, Mutah Faculty of Medicine, Mutah University, P.O. Box 7 Mutah, Alkarak 61710, Jordan; hala.altarawneh@mutah.edu.jo; 2 Department of Medical Microbiology and Immunology, Faculty of Medicine, Menoufia University, Shebin El-Kom 32511, Menoufia, Egypt; eman.hosney.salem@med.menofia.edu.eg; 3 Department of Biochemistry, Shebin El-Kom Teaching Hospital, Shebin El-Kom 32511, Menoufia, Egypt; asmaamohamedsoliman93@gmail.com; 4 Department of Clinical Pharmacology, Faculty of Medicine, Menoufia University, Shebin El-Kom 32511, Menoufia, Egypt; amany.elfakhrany@med.menofia.edu.eg; 5 Department of Medical Biochemistry and Molecular Biology, College of Medicine, Al-Nahada Colleges of Pharmacy and Medical Sciences, Riyadh P.O. Box 13253, Saudi Arabia; heba.ali@fmed.bu.edu.eg; 6 Department of Medical Biochemistry and Molecular Biology, Benha Faculty of Medicine, Benha University, Benha 13518, Egypt; 7 Clinical Pathology, National Liver Institute, Menoufia University, Shebin El-Kom 32511, Menoufia, Egypt; yasmin.mohsen@liver.menofia.edu.eg; 8 Clinical Pathology Department, National Cancer Institute, Cairo University, Cairo 11796, Egypt; abdallah.mohamed@nci.cu.edu.eg; 9 Department of Clinical Microbiology and Immunology, National Liver Institute, Menoufia University, Shebin El-Kom 32511, Menoufia, Egypt; halabazeed@gmail.com

**Keywords:** *Candida* species, virulence genes, immunocompromised patients, antifungal susceptibility, real-time PCR

## Abstract

The incidence of fungal infections, particularly those caused by *Candida* species, has significantly risen among immunocompromised patients in recent years. **Aim:** This study evaluated the species distribution, antifungal susceptibility patterns, and virulence gene profiles of Candida species isolated from critically ill ICU patients while comparing the diagnostic performance of conventional phenotypic isolation media with molecular genotypic identification methods. **Materials and Methods:** A total of 211 clinical specimens were obtained from 200 hospitalized patients at Menoufia University Hospitals, Egypt. *Candida* species were phenotypically identified using cornmeal agar supplemented with Tween 80 (CTA) and CHROMagar^TM^ *Candida* (CMA) and were molecularly confirmed at the species level by sequencing PCR-amplified *ITS* regions. Antifungal susceptibility profiles to fluconazole, voriconazole, and amphotericin B were evaluated using the disk diffusion method. Furthermore, real-time PCR was deployed to detect genes encoding adherence factors and Secreted Aspartyl Proteinase. **Results:** *Candida* species were recovered from 45 out of 211 clinical specimens. The most frequently isolated species was *Candida albicans* (15/45, 33.3%), followed by *C. parapsilosis* (9/45, 20.0%), *C. glabrata* (7/45, 15.5%), *C. tropicalis* (7/45, 15.5%), *C. krusei* (4/45, 8.8%), and *C. famata* (3/45, 6.7%). Among the recovered *Candida* isolates, the percentage rates of the *ALS1*, *ALS3*, *HWP1*, and *SAP4* genes were 40%, 42.22%, 44.4%, and 37.78%, respectively. *HWP1* was the most frequently detected pathogenicity factor, whereas *SAP4* was the least prevalent. **Conclusions:** In the studied immunocompromised cohort, non-*albicans Candida* species constituted a substantial proportion of clinical isolates. Notable antifungal resistance patterns were also observed, emphasizing the value of integrated phenotypic and genotypic identification for tailored management. Furthermore, virulence-associated genes (*ALS1*, *ALS3*, *HWP1*, and *SAP4*) were detected in isolates of several *Candida* species, highlighting their presence in clinical strains.

## 1. Introduction

Candida species are well-recognized components of the commensal human microbiota, colonizing the skin, oral cavity, gastrointestinal tract, and genitourinary membranes [1]. However, they act as opportunistic pathogens and are capable of causing a wide spectrum of infections, particularly in immunocompromised patients, ranging from superficial mucosal lesions to life-threatening invasive diseases. The clinical term “invasive candidiasis” encompasses candidemia as well as deep-seated infections of normally sterile body sites [2]. Notably, over 90% of invasive candidiasis cases are attributed to five main pathogens—*Candida albicans*, *Candida glabrata* (*Nakaseomyces glabratus*), *Candida krusei* (*Pichia kudriavzevii*), *Candida tropicalis*, and *Candida parapsilosis*—with candidemia being the most frequently identified manifestation [3]. Although the prevalence of non-albicans Candida (NAC) species has been steadily rising globally, *C. albicans* remains the predominant etiologic agent of these infections [4]. These opportunistic yeasts pose a severe threat to hospitalized patients, especially those with debilitating underlying conditions, making targeted antifungal therapy a cornerstone of clinical management [5].

Nevertheless, the extensive and prolonged clinical deployment of antifungal agents has inevitably led to decreased drug sensitivity and the rapid emergence of resistant *Candida* strains. Consequently, continuous surveillance of the shifting distribution of *Candida* species and their corresponding antifungal resistance patterns is crucial for optimizing patient management. Such monitoring programs provide indispensable data that guide prophylactic strategies, empirical therapies, and the effective treatment of severe candidiasis [6].

Currently, several antifungal classes are available to combat *Candida* infections, including polyenes, azoles, allylamines, and echinocandins. The clinical efficacy of these agents varies substantially depending on the type and anatomical location of the infection, as well as the specific susceptibility profiles of the infecting *Candida* species. Due to their high success rates and efficacy even during short therapeutic courses, azole antifungals are among the most frequently prescribed treatments. However, this escalating usage has driven the development of resistance among previously susceptible *Candida* populations [7]. To counter this, standardized antifungal susceptibility testing methodologies—such as broth microdilution, disk diffusion, and E-testing—are now widely utilized. These approaches rely on species-specific clinical breakpoints established and updated by the Clinical and Laboratory Standards Institute (CLSI) [8].

Concurrently, the taxonomy of yeasts and other fungi has undergone significant refinement since the integration of DNA sequencing into microbial classification. Modern identification of yeast species frequently involves sequencing single polymorphic gene targets, such as the internal transcribed spacer (*ITS*) regions or the D1/D2 domain of the 26S rDNA. In fact, the *ITS* region sequence has been officially proposed as the primary fungal barcode marker by the Consortium for the Barcode of Life [9].

The propensity of certain *Candida* species to cause disseminated, invasive infections is closely linked to their arsenal of virulence factors. *C. albicans*, *C. tropicalis*, and *C. glabrata* are recognized as the most virulent species, with their infections associated with higher mortality rates. *Candida* pathogenicity is driven by multifaceted mechanisms that facilitate adherence to target host cells, tissue invasion, biofilm formation, yeast-to-hyphae morphological transition, evasion of the host immune response, and the secretion of tissue-damaging hydrolytic enzymes, including phospholipases, proteases, and hemolysins [10].

Among these virulence determinants, the Agglutinin-Like Sequence family (including *ALS1* and *ALS3*) and Hyphal Wall Protein 1 (*HWP1*) play pivotal roles in fungal adherence to host tissues and subsequent biofilm development, actions that severely restrict antifungal drug penetration [11,12]. Additionally, Secreted Aspartyl Proteinases (such as *SAP4*) facilitate host tissue degradation and active immune evasion [13]. Investigating the distribution of these specific virulence genes is of paramount clinical importance, not only for decoding species-specific pathogenicity but also for exploring their potential contribution to biofilm-associated antifungal resistance, which further complicates treatment outcomes in immunocompromised cohorts [11,12].

The novelty of the present study lies in its comprehensive approach, combining phenotypic identification, antifungal susceptibility testing, and molecular detection of key virulence genes among various *Candida* species recovered from immunocompromised patients at Menoufia University Hospitals, Egypt. The objective of this study was to investigate the species distribution, antifungal susceptibility patterns, and virulence gene profiles of *Candida* species isolated from critically ill ICU patients. An additional aim was to evaluate the diagnostic accuracy and concordance of conventional phenotypic identification media in comparison with molecular identification methods.

## 2. Materials and Methods

### 2.1. Study Design

This prospective cross-sectional study was conducted across the Departments of Medical Microbiology (Faculty of Medicine, Menoufia University, Shebin El-Kom, Egypt) and Clinical Pathology (Shebin El-Kom Teaching Hospitals), Shebin El-Kom, Egypt. The study cohort comprised 200 immunocompromised patients (111 males and 89 females) admitted to various specialized units—including the neonatal, renal, and hepatic intensive care units (ICUs), as well as burn and diabetes mellitus units—at Menoufia University Hospitals between 1 March 2024, and 31 August 2024. Inclusion Criteria: Patients were classified as immunocompromised and included in the study based on the following established clinical criteria: 1. Hematological Malignancies: Patients diagnosed with active lymphoma or leukemia. 2. Solid Organ Transplantation: Recipients of solid organ transplants currently undergoing active immunosuppressive regimens. 3. Chronic Comorbidities: Patients presenting with end-stage renal disease (ESRD), advanced hepatic illness, or uncontrolled diabetes mellitus. 4. Pharmacological Immunosuppression: Individuals who received chemotherapy or systemic corticosteroid therapy (e.g., prednisolone > 20 mg/day for >2 weeks) within the three months prior to enrollment. 5. Neonatal Status: Critically ill neonates admitted to the neonatal ICU (NICU). Exclusion Criteria: 1. Patients with an ICU stay duration of less than 48 h. 2. Immunocompetent individuals lacking underlying immunosuppressive conditions or chronic systemic disorders. 3. Patients who had received systemic antifungal therapy or antifungal prophylaxis prior to specimen collection. 4. Patients who declined to provide informed written consent for participation or whose legal guardians declined to provide consent.

### 2.2. Specimen Collection and Processing

Clinical specimens—including blood, cerebrospinal fluid (CSF), urine, Foley catheter tips, sputum, and bronchoalveolar lavage (BAL) fluid—were collected under strict aseptic precautions from patients exhibiting clear clinical signs and symptoms of infection (excluding any asymptomatic or pure colonization cases). All specimens were initially inoculated onto blood agar and Sabouraud Dextrose Agar (SDA). For fungal isolation, samples were cultured on SDA medium supplemented with chloramphenicol (0.5 g/L; Oxoid, Basingstoke, UK) and incubated at both 37 °C and 25 °C for 48–72 h. Growing colonies were subsequently processed using conventional mycological methods.

For respiratory specimens (sputum and BAL), differentiation between *Candida* colonization and true infection was established based on clinical manifestations (including fever, purulent secretions, and new radiologic infiltrates) paired with quantitative culture results, utilizing a threshold of ≥10^4^ CFU/mL for BAL to signify infection. However, positive respiratory cultures were interpreted with caution; even in symptomatic patients with significant quantitative counts >10^4^ CFU/mL, *Candida* is frequently regarded as a respiratory commensal and may not act as the primary etiological driver of lower respiratory tract infections, as extensively reviewed by Pendleton et al. (2017) [14]. Similarly, *Candida* strains isolated from Foley catheter tips were only included from symptomatic patients with clinically suspected urinary tract infections (UTIs) in whom no concurrent bacterial growth was detected.

### 2.3. Phenotypic Identification and Antifungal Susceptibility Testing

Fungal growth on SDA was monitored to observe macro-morphological characteristics. Gram staining, the germ tube test, and subsequent sub-culturing on specialized morphology media—including cornmeal agar supplemented with Tween 80 (CTA) and CHROMagar^TM^ *Candida* (CMA; Paris, France)—were employed for the presumptive identification and differential screening of various *Candida* species [7]. To eliminate subjectivity inherent to phenotypic assays and ensure the definitive identification of cryptic or rare species (such as *C. famata*), the 45 *Candida* isolates were subjected to downstream molecular confirmation via PCR and sequencing.

Antifungal Susceptibility Testing (Disk Diffusion) for fluconazole, voriconazole, amphotericin B, and micafungin: In vitro antifungal susceptibility profiles were determined using the disk diffusion method. Zone diameters were interpreted in accordance with the Clinical and Laboratory Standards Institute (CLSI) guidelines, documents M44 (3rd edition) and M27 (4th edition) (CLSI, 2022) for fluconazole, voriconazole, and micafungin [8]. For amphotericin B, disk diffusion testing was performed for preliminary qualitative screening and the results were evaluated according to the manufacturer’s instructions and published interpretive guidance for *Candida* disk diffusion testing (Espinel-Ingroff et al., 2007) [15], as CLSI clinical breakpoints have not been formally established for disk diffusion of polyene. The isolates were screened against fluconazole (25 µg), Voriconazole (1 µg), Amphotericin B (100 Units) (Oxoid, Basingstoke, UK), and micafungin (2 µg) (Oxoid, UK). Inocula were prepared by suspending distinct colonies in sterile physiological saline to match a 0.5 McFarland turbidity standard, followed by lawn inoculation onto Mueller–Hinton agar (MHA) supplemented with 2% glucose and 0.5 µg/mL methylene blue. Inhibition zones were measured following incubation at 37 °C for 24–48 h. Quality control was ensured using the reference strains *Candida albicans* ATCC 90028 and *Candida krusei* ATCC 6258.

Antifungal Susceptibility Testing (E-test) for fluconazole and voriconazole: Minimum Inhibitory Concentrations (MICs) were determined using the Epsilometer test (E-test; Oxoid, Basingstoke, UK) in strict accordance with the manufacturer’s protocols. Each microbial suspension (adjusted to a 0.5 McFarland standard) was inoculated onto MHA supplemented with 2% glucose and 0.5 µg/mL methylene blue. Following the application of specific E-test strips (fluconazole and voriconazole) and subsequent incubation at 37 °C for 24–48 h, the MIC values were read at the point of complete inhibition. Results were interpreted based on the defined CLSI clinical breakpoints (CLSI, 2022) [8], except for species lacking formal clinical breakpoints. Specifically, for Candida glabrata and Candida famata, interpretation was performed using Epidemiological Cutoff Values (ECVs), categorizing isolates into wild-type (WT) and non-wild-type (non-WT) populations rather than clinical susceptibility categories.

### 2.4. Genotypic Confirmation of Candida Isolates

#### 2.4.1. DNA Extraction

Genomic DNA was extracted from log-phase *Candida* colonies cultivated on CHROMagar^TM^ medium using the QIAamp DNA Mini Kit (Qiagen, Hilden, Germany) following the manufacturer’s spin-column protocol. The purified DNA was quantified and stored at −80 °C until molecular analysis.

#### 2.4.2. PCR Amplification of the Internal Transcribed Spacer (ITS) Regions

The complete rDNA ITS region was amplified using the fungus-specific universal primer pair: ITS1: (5′-TCCGTAGGTGAACCTGCGG-3′); ITS4: (5′-TCCTCCGCTTATTGATATGC-3′) [16].

All 45 clinical isolates were subjected to PCR amplification using a thermal cycler (Biometra, Göttingen, Germany). The PCR amplifications were performed in a total volume of 50 μL. Each reaction mixture contained 1X reaction buffer (10 mM Tris-HCL (pH 8.4), 50 mM of KCl, 1.5 mM of MgCl_2_), 5% DMSO, 0.2 mM of dATP, dCTP, dGTP, and dTTP (Fermentas International, Inc., Burlington, ON, Canada), 0.1 μg of each of the primers (*ITS*1 and *ITS*4), 1.25 U of TaqDNA polymerase (Invitrogen-Life Technologies, São Paulo, Brazil), and 30 ng of fungal genomic DNA. The amplification profile consisted of an initial denaturation step at 95 °C for 7 min, followed by 40 cycles of denaturation at 95 °C for 1 min, primer annealing at 54 °C for 2 min, and extension at 72 °C for 1 min, with a final extension step at 72 °C for 10 min [15]. The resulting PCR products were resolved via horizontal gel electrophoresis on a 1.5% (*w*/*v*) agarose gel stained with ethidium bromide (0.5 μg /mL). Electrophoresis was performed in 1X TBE buffer at 80–100 V for approximately 45–60 min. A 100 bp DNA ladder was loaded alongside the samples to determine amplicon sizes, which ranged between 500 and 550 bp depending on the species. DNA bands were visualized and digitally photographed under a UV transilluminator (Gel Documentation System).

#### 2.4.3. Sanger Sequencing and Bioinformatic Analysis

A further sequencing step was applied to the 45 *Candida* isolates to genetically validate species identity and compare traditional morphological findings against the molecular gold standard [17]. Prior to sequencing, the amplified PCR products were purified using a commercial spin-column kit (e.g., QIAquick PCR Purification Kit, Qiagen, Hilden, Germany) according to the manufacturer’s instructions to eliminate PCR contaminants, primers, and unreacted dNTPs. Direct sequencing was performed using Taq Dye Deoxy and ABI Prism terminator cycle sequencing kits (Applied Biosystems, Foster City, CA, USA) utilizing the original ITS1 and ITS4 primers. The obtained nucleotide sequences were analyzed and aligned using the Basic Local Alignment Search Tool (BLASTn) against reference sequences on the NCBI database (http://www.ncbi.nlm.nih.gov) (accessed on 15 May 2025) to identify species definitively based on maximal sequence homology [18]. Species assignment required a sequence identity of ≥99% and a query coverage of ≥98% against reference strains. Due to high sequence conservation among the isolates, only three representative sequences for *C. albicans*, *C. tropicalis*, and *C. paapsilosis* were successfully obtained and deposited in the GenBank database under accession numbers (KP675291.1), (OM523874.1), and (KP674500.1), respectively.

### 2.5. Molecular Detection of Virulence Genes Using Real-Time PCR

Real-time PCR was deployed for the molecular screening of key virulence determinants, specifically the *ALS1*, *ALS3*, *HWP1*, and *SAP4* genes, among the confirmed *Candida* isolates. The gene-specific primer sequences utilized for real-time PCR profiling are summarized in Table 1 [19].

Real-time PCR amplification and detection were performed using the Applied Biosystems 7500 Real-Time PCR System. A specific reaction mixture was prepared for each targeted gene in a separate well, with a final reaction volume of 25 μL. Each reaction contained 12.5 μL of Maxima SYBR Green qPCR Master Mix, 1 μL of forward primer, 1 μL of reverse primer, 5 μL of extracted *Candida* template DNA, and 5.5 μL of nuclease-free water.

The optimized cycling profile consisted of an initial denaturation step at 95 °C for 10 min (1 cycle), followed by 40 cycles of a three-stage thermal profile: denaturation at 95 °C for 15 s, primer annealing at 60 °C for 30 s, and extension at 72 °C for 30 s. To guarantee assay accuracy and specificity, nuclease-free water was included as a negative control, while genomic DNA extracted from *Candida* albicans ATCC 90028 served as the positive control. Following amplification, a post-PCR melt curve analysis was systematically carried out to confirm the specificity of the amplified products. The absence of primer dimers or non-specific amplification products was verified by the detection of a single, sharp derivative peak at the characteristic melting temperature (Tm) unique to each gene. Gene positivity was determined based on the cycle threshold (Ct) values, where Ct ≤ 35 was defined as positive and Ct > 35 was considered negative.

### 2.6. Ethical Considerations and Consent to Participate

Ethical approval was granted by the Institutional Ethics Committee of the Faculty of Medicine, Menoufia University (approval reference number: 1/2024MICR6), and informed written consent was obtained from all patients or their legal guardians prior to enrollment.

### 2.7. Statistical Analysis

Data were analyzed using SPSS, version 26 (IBM Corp., Armonk, NY, USA). Categorical variables are presented as numbers and percentages. Associations between categorical variables were evaluated using Pearson’s Chi-square test, or Fisher’s exact test when expected cell counts were less than 5. To estimate the strength of association for predisposing factors, Odds Ratios (ORs) with their corresponding 95% Confidence Intervals (95% CIs) were calculated. Cohen’s kappa coefficient was used to measure inter-method agreement according to the following standard scale: kappa < 0: no agreement; kappa 0–0.20: slight agreement; kappa 0.21–0.40: fair agreement; kappa 0.41–0.60: moderate agreement; kappa 0.61–0.80: substantial agreement; and kappa 0.81–1.00: almost perfect agreement. All assumptions for the utilized tests were verified. Prevalence estimates and key percentage metrics are accompanied by their respective 95% CIs. Statistical significance was set at a *p*-value of less than 0.05 [20].

## 3. Results

### 3.1. Demographic and Clinical Characteristics of the Cohort

For the present study, a total of 211 clinical specimens were retrieved from 200 immunocompromised patients. The gender distribution of the cohort exhibited a higher prevalence of males (n = 111, 55.5%) compared to females (*n* = 89, 44.5%), with a mean patient age of 41 years. Among the collected specimens, blood cultures constituted the majority (*n* = 121, 57.35%), followed by urine and Foley catheter tips (*n* = 44, 20.8%), sputum and bronchoalveolar lavage (BAL) fluids (*n* = 40, 19.0%), and cerebrospinal fluid (CSF) samples (*n* = 6, 2.85%).

Of the 211 processed samples, 48 (22.7%) exhibited no microbiological growth after a standard 48-h incubation period, whereas 163 samples (77.3%) demonstrated positive microbiological growth. Among the culture-positive samples, pure bacterial infections were the most prevalent, accounting for 65.6% (107/163), followed by pure *Candida* infections at 20.2% (33/163) and mixed bacterial–*Candidal* co-infections at 7.3% (12/163). Additionally, *Aspergillus* species were isolated from 6.7% (11/163) of the positive samples (Supplementary Data, Table S1). Statistical analysis demonstrated that the empirical and prolonged misuse of broad-spectrum antibiotics, uncontrolled diabetes mellitus, parenteral nutrition, indwelling urinary catheterization, neonatal status, and central venous catheterization (CVC) were statistically significant risk factors predisposing patients to *Candida* infections and candidemia (Supplementary Data, Table S2).

### 3.2. Performance of Phenotypic Media Versus Genotypic Confirmation

**Figure 1:**
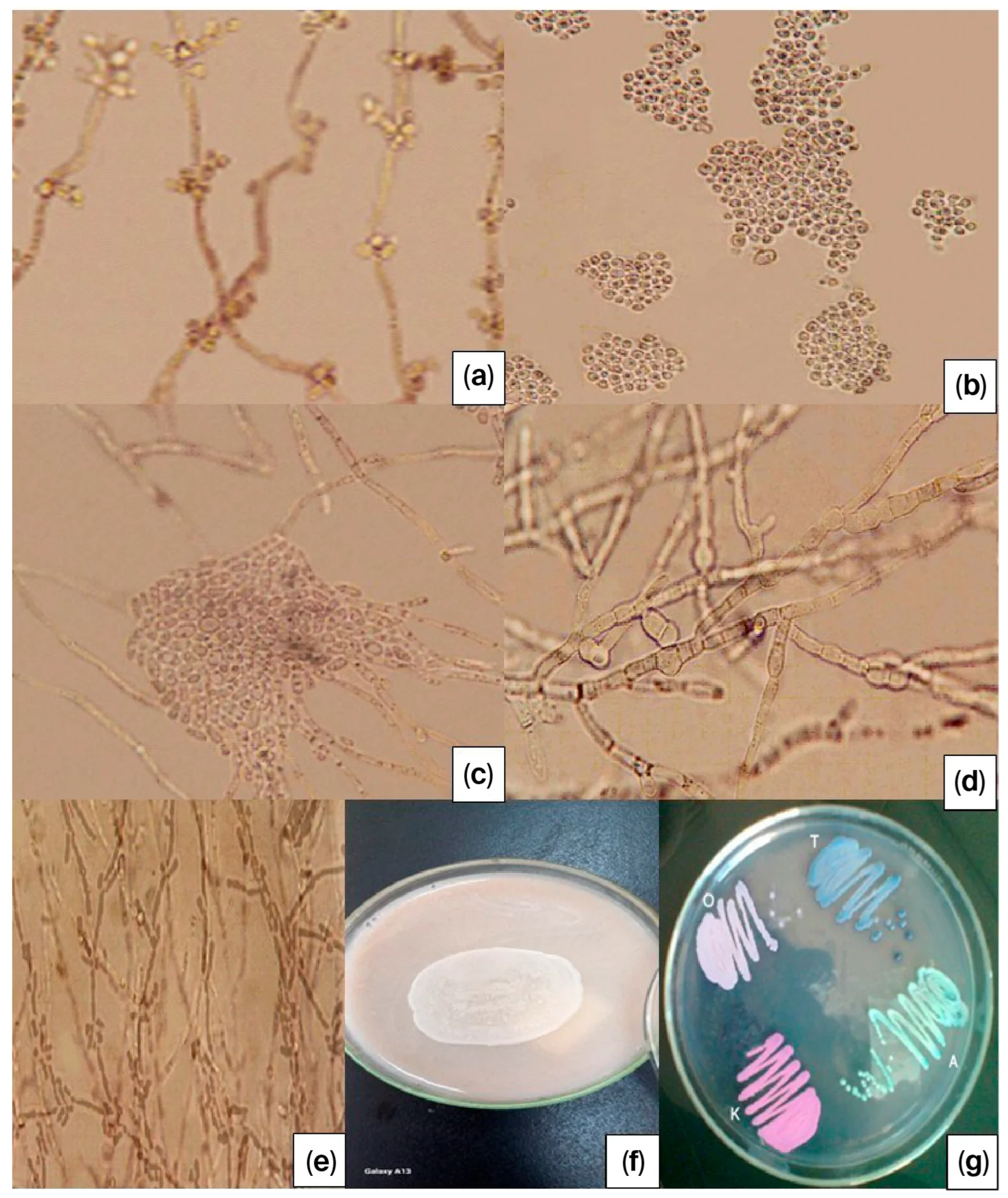
Growth characteristics of isolated *Candida* species on culture media. (**a**) Microscopic appearance of *C. albicans* on cornmeal agar–Tween 80 showing chlamydospores, which are round, thick-walled structures, often found terminally on pseudohyphae. (**b**) Microscopic appearance of *C. glabrata* on cornmeal agar–Tween 80 showing small, smooth, creamy-white yeast colonies, lacking pseudohyphae. (**c**) Microscopic appearance of *C. tropicalis* on cornmeal agar–Tween 80 showing abundant, branching pseudohyphae, with oval blastospores (budding yeast cells) appearing along the pseudohyphae. (**d**) Microscopic appearance of *C. parapsilosis* on cornmeal agar–Tween 80 showing blastospores along pseudohyphae. (**e**) Microscopic appearance of *C. krusei on* cornmeal agar–Tween 80 showing elongated blastoconidia (up to 25 µm long) with a “matchstick” appearance. (**f**) *C. famata* on CHROMagar^TM^ showing light pink colonies. (**g**) *Candida* species growth on CHROM agar: (*A*) *C. albicans* produced green colonies, (T) *C. tropicalis* produced blue colonies, (K) *C. krusei* produced pinkish purple colonies, and (*O*) *C. glabrata* produced characteristic dark violet to purple colonies on CHROMagar^TM^.

Both cornmeal agar supplemented with Tween 80 (CTA) and CHROMagar^TM^ *Candida* (CMA) successfully facilitated the identification of *C. albicans*, *C. tropicalis*, and *C. krusei*. Differential identification of *C. glabrata* and *C. parapsilosis* was achievable on CTA but not on CMA (Figure 1).

**Figure 2:**
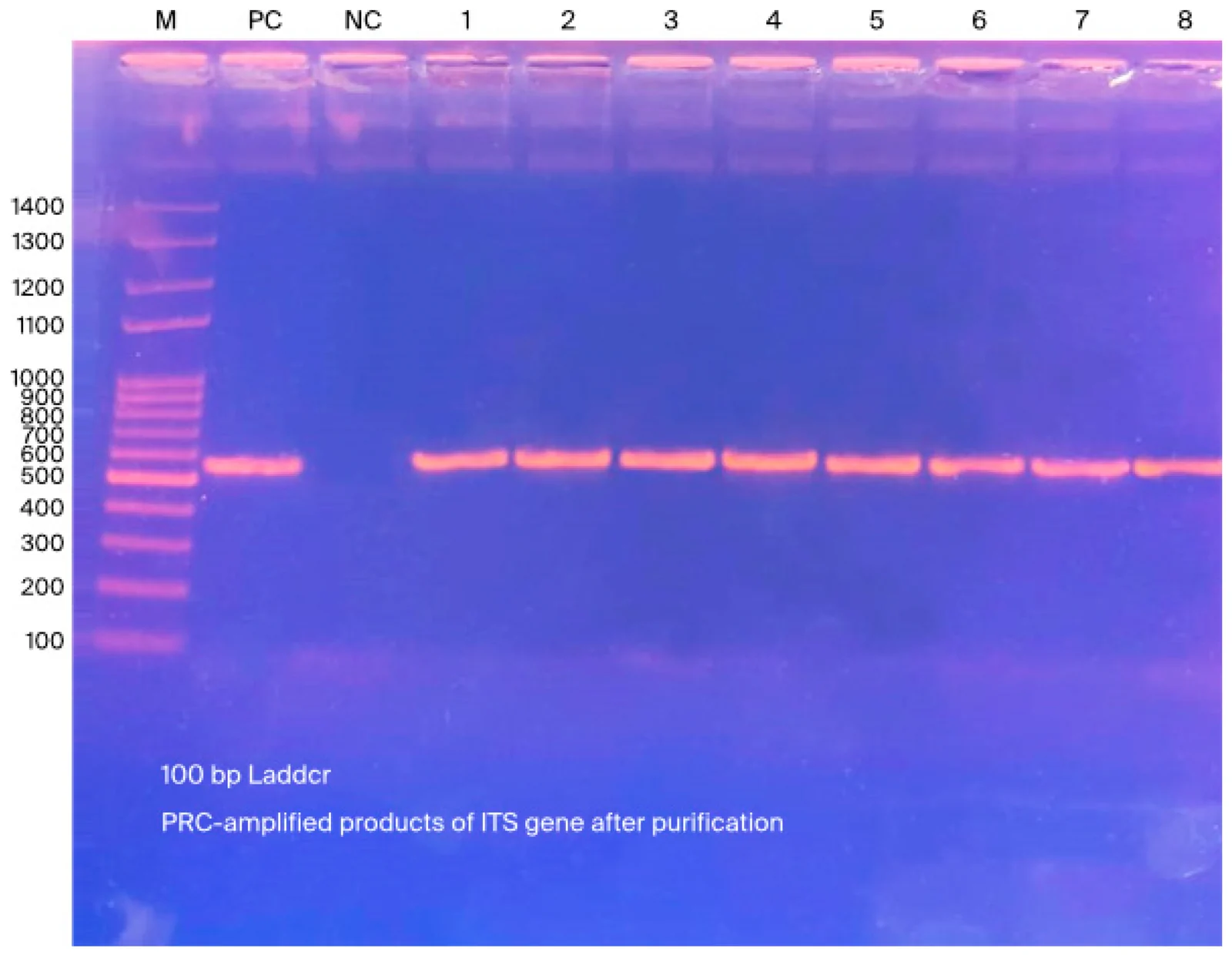
Agarose gel electrophoresis for the PCR-amplified products of the ITS gene after purification. Lane M: 100 bp DNA ladder (molecular weight marker); Lane PC: positive control (PC) *Candida albicans* ATCC 90028; Lane NC: negative control (NC); Lanes 1–8: Clinical *Candida* isolates showing specific amplified bands at the expected size range of 500–550 bp. N.B: lane 1: *Candida albicans*, lane 2: *C. parapsiliosis*, lane 3: *C. famata*, lane 4: *C. tropicalis*, lane 5: *C. krusei*, lane 6: *C. glabrata*, lane 7: *C. parapsiliosis*, lane 8: *C. albicans*.

To definitively validate species identity, internal transcribed spacer (ITS) rDNA PCR was deployed for 45 presumptive *Candida* isolates, yielding a 100% amplification success rate for all culture-positive isolates (Figure 2). Moreover, direct Sanger sequencing of representative PCR products from each identified *Candida* species demonstrated a 100% confirmation and correlation rate with the genotypic PCR bands.

*Candida* albicans was identified as the most frequently isolated species, accounting for 33.3% (15/45) of the total isolates, followed by *C. parapsilosis* (*n* = 9, 20.0%), *C. glabrata* (*n* = 7, 15.5%), *C. tropicalis* (*n* = 7, 15.5%), *C. krusei* (*n* = 4, 8.8%), and *C. famata* (*n* = 3, 6.7%) (Table 2).

Comparative analysis between CHROMagar™ and the molecular gold standard (sequencing) revealed that CHROMagar™ achieved 100% sensitivity, specificity, positive predictive value (PPV), and negative predictive value (NPV) when identifying *C. albicans*, *C. tropicalis*, and *C. krusei*. However, for *C. famata*, while sensitivity, NPV, and specificity reached 100%, 100%, and 95.24% respectively, its positive predictive value (PPV) dropped to 60% due to the misidentification of two *C. parapsilosis* isolates (Table 3). Conversely, CTA demonstrated 100% sensitivity and specificity when detecting *C. glabrata*, but failed to accurately diagnose 22.2% (2/9) of *C. parapsilosis* isolates and all of the *C. famata* isolates (Table 4).

### 3.3. Antifungal Susceptibility Testing Profiles

Based on the disk diffusion method (Table 5), the 45 confirmed *Candida* isolates exhibited the highest resistance rates against fluconazole (15/45, 33.33%) and voriconazole (5/45, 11.11%). Conversely, the highest susceptibility rates were observed against Amphotericin B (44/45, 97.78%), followed closely by Micafungin (42/45, 93.33%). Species-specific resistance patterns revealed that only one strain of *C. parapsilosis* and two strains of *C. famata* displayed resistance to Micafungin, while a single strain of *C. glabrata* was resistant to Amphotericin B (Table 5).

**Figure 3:**
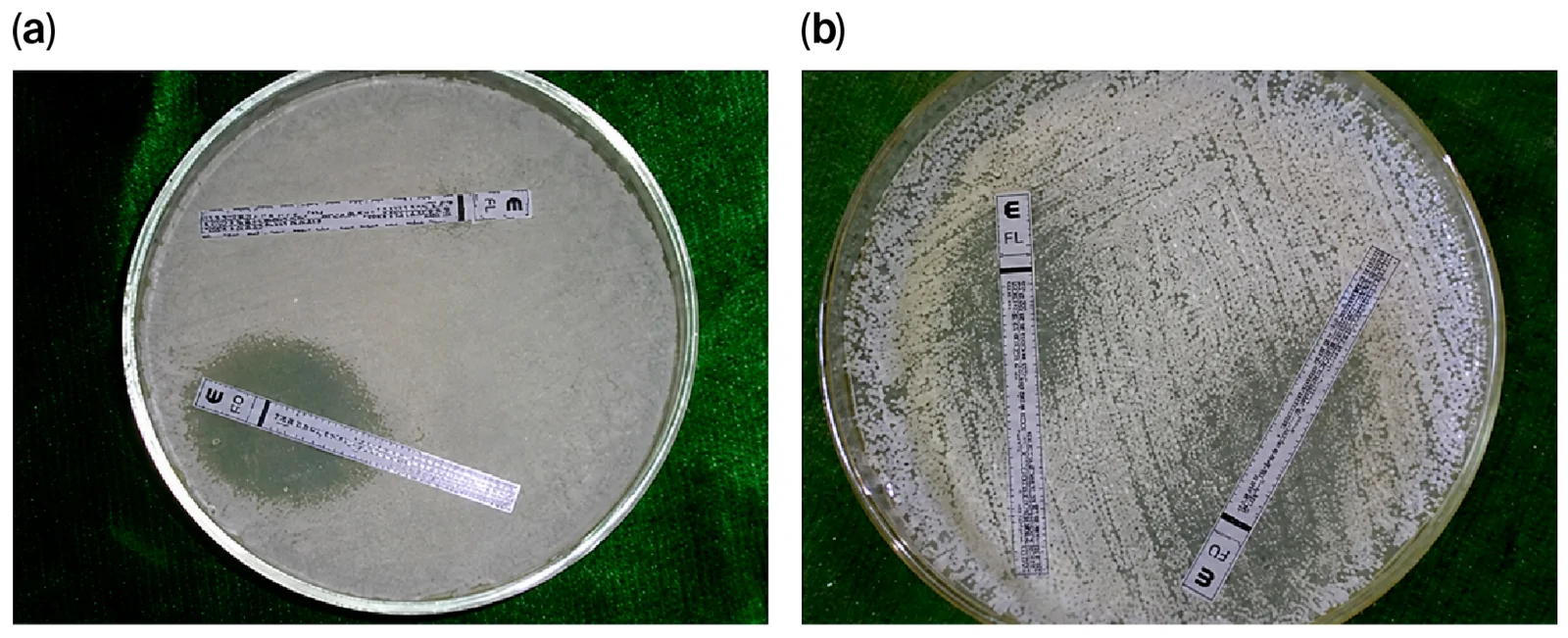
E-test gradient diffusion method on Mueller–Hinton agar with 2% glucose for antifungal susceptibility testing. (**a**): *Candida albicans* isolate demonstrating sensitivity to voriconazole (ellipse intersection at MIC = 0.032 µg/mL) and resistance to fluconazole (MIC > 8 µg/mL). (**b**): *Candida tropicalis* isolate exhibiting multi-drug resistance with uninhibited growth up to the highest concentrations of fluconazole (MIC > 8 µg/mL) and voriconazole (MIC ≥ 1 µg/mL), as evidenced by the inhibition ellipses intersecting at elevated MIC endpoints on the test strips.

To precisely determine the Minimum Inhibitory Concentrations (MICs), the E-test methodology was utilized (Table 6 and Table 7, Figure 3). For fluconazole (Table 6), 48.89% (22/45) of the tested strains exhibited resistance, while 17.78% (8/45) demonstrated susceptible-dose dependent (S-DD) status, and 33.33% (15/45) exhibited sensitivity (Table 6). Regarding voriconazole (Table 7), 6.67% (3/45) of the total strains were confirmed as resistant, while 53.33% (22/45) exhibited sensitivity and 17.78% (8/45) demonstrated a susceptible-dose dependent (S-DD) profile. Regarding *Candida glabrata* isolates (*n* = 7), as formal CLSI clinical breakpoints are lacking, interpretation against voriconazole was performed using the epidemiological cutoff value (ECV) of 0.5 mcg/mL. Out of the 7 tested isolates, 5 (71.43%) exhibited MICs ≤ 0.5 mcg/mL and were classified as wild-type (WT), while 2 (28.57%) exhibited MICs > 0.5 mcg/mL and were categorized as non-wild-type (non-WT).

### 3.4. Prevalence and Comparative Analysis of Virulence Genes

The distribution and comparative profiling results of the investigated virulence determinants (*ALS1*, *ALS3*, *HWP1*, and *SAP4*) across the 45 *Candida* isolates are summarized in Table 8. *HWP1* was the most prevalent virulence gene detected among all isolates (44.44%), followed closely by *ALS3* (42.22%) and *ALS1* (40.0%), whereas *SAP4* was the least frequently detected (37.77%).

**Figure 4:**
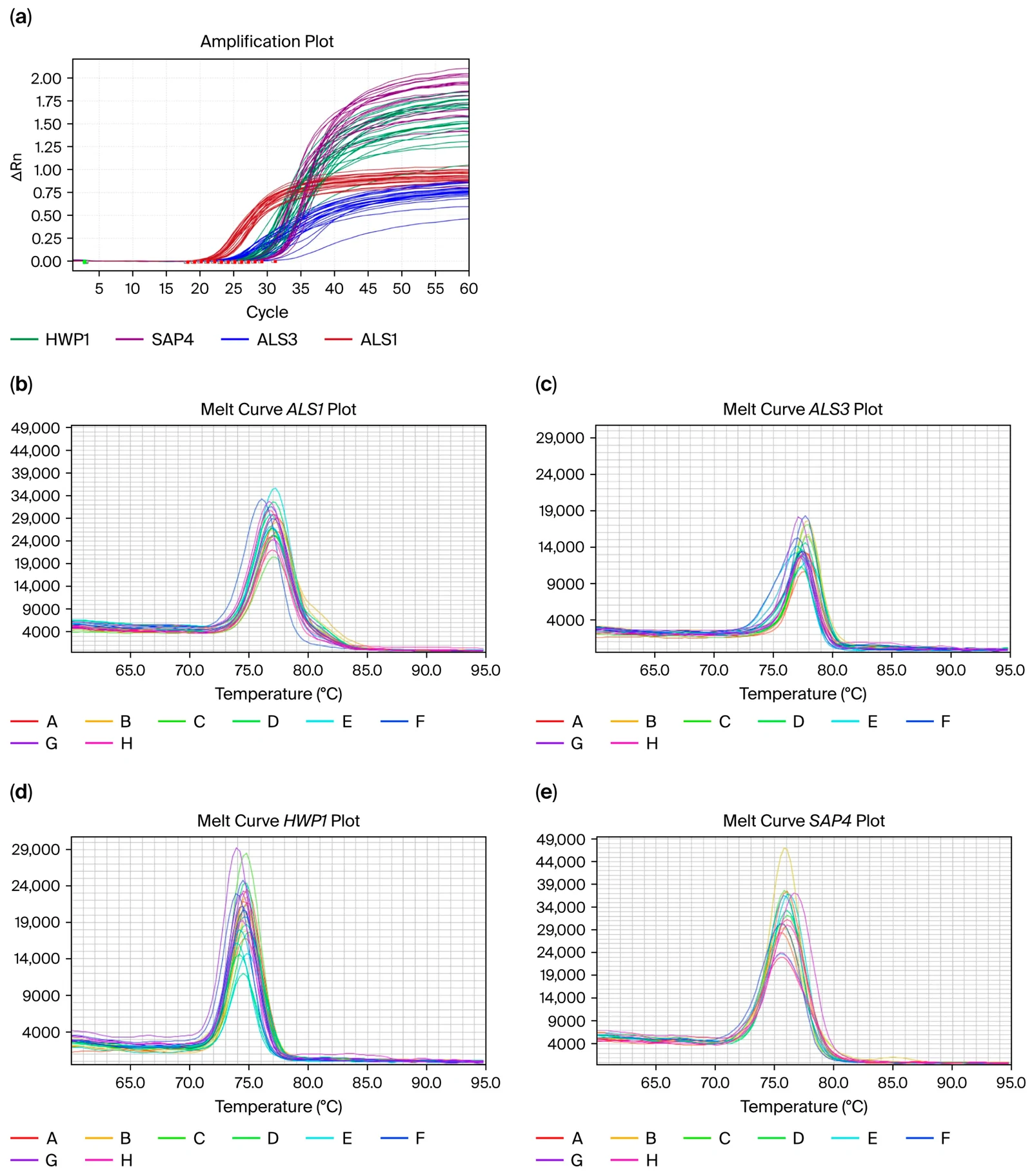
Amplification plots and melting curve analysis for the target Candida virulence genes (*HWP1*, *SAP4*, *ALS1*, and *ALS3*). (**a**): Representative real-time PCR amplification plot showing log-phase curves for the target genes. (**b**): Melting curve profile of *ALS1* gene with a specific melting temperature Tm at approximately 77.0 °C. (**c**): Melting curve profile of *ALS3* gene with a specific Tm at approximately 78.0 °C. (**d**): Melting curve profile of *HWP1* gene with a specific Tm at approximately 76.5 °C. (**e**): Melting curve profile of *SAP4* gene with a specific Tm at approximately 77.5 °C. The single sharp dissociation peaks confirm reaction specificity without non-specific products or primer dimers.

Notably, all studied virulence genes exhibited a significantly higher frequency in *C. albicans* isolates compared to non-*albicans Candida* species (*p* < 0.001 for *ALS1*, *ALS3*, and *HWP1*; *p* = 0.001 for *SAP4*; Fisher’s exact test). For instance, *HWP1* positivity was observed in 93.33% of *C. albicans* isolates, compared to 57.14% of *C. glabrata* and 28.6% of *C. tropicalis*. Furthermore, while *ALS1* and *ALS3* were often detected in *C. albicans* (86.7%), their detection rate dropped to 0% for ALS1 in both *C. tropicalis* and *C. krusei* and 14.3% for *ALS3*. The *SAP4* gene was detected in 73.33% of *C. albicans* isolates and notably stood out as the sole virulence gene identified among *C. famata* isolates, with a positivity rate of 33.33% (Table 8, Figure 4).

## 4. Discussion

*Candida albicans* remains the most common etiology of fungal infections globally; however, non-*albicans Candida* (NC) species represent a significant proportion of clinical isolates [21]. Accurately identifying distinct *Candida* species is clinically vital for optimizing the selection of appropriate antifungal therapies [22]. Although a diverse array of antifungal classes are currently utilized to manage these mycoses, the escalating clinical deployment of these drugs has driven the emergence of resistance among previously susceptible *Candida* populations [7]. In the present study, 211 clinical specimens were recovered from 200 patients with suspected candidiasis admitted to the ICUs of Menoufia University Hospitals and Shebin El-Kom Teaching Hospitals. Of these, 45 specimens yielded positive *Candida* growth. Following phenotypic screening and definitive confirmation via PCR and sequencing, *C. albicans* accounted for 33.33% (15/45) of the isolates, whereas non-*albicans Candida* species predominated at 66.67% (30/45). These findings closely align with those reported by El-Sayed and El-Khayat [23], Daef et al. [24], and Aboueldahab et al. [25]. Collectively, these data support the well-documented global epidemiological shift in Candida infections over the last few decades toward non-*albicans* species. In contrast, a lower prevalence of non-*albicans Candida* isolates was previously recorded by Kadry et al. (42.6%) [26] and El-Ganiny et al. (29.3%) [27]. Among the recovered isolates in our cohort, *C. albicans* was the most prevalent (33.3%), followed by *C. parapsilosis* (20.0%). Conversely, *C. famata* exhibited the lowest prevalence (6.7%), followed by *C. krusei* (8.9%). These distribution patterns are highly consistent with findings documented by Ardehali et al. [19], Klingspor [28], Zhu [29], and Roudbary et al. [30].

To accurately differentiate true lower respiratory tract *Candida* infection from upper airway oral colonization, strict cytological and microscopic evaluations of sputum samples were performed. Specimens demonstrating low squamous epithelial cell counts (<10/LPF) and high polymorphonuclear leukocyte (PMN) counts (>25/LPF), indicating a genuine pulmonary infection rather than salivary contamination, were selectively processed [30]. This prominent PMN infiltration, combined with manifest clinical signs, strongly underscores the pathogenic role of the isolated *Candida* strains in these patients. Furthermore, 14 *Candida*-positive blood cultures were recovered in our study; *C. albicans* was identified in 9 (64.3%) of these samples. This high prevalence of *C. albicans* in fungemia is consistent with the results of prior investigations conducted by Pfaller et al., Rodero et al., Sandven et al., Alvarado et al., and Rahbar et al. [31,32,33,34,35]. The remaining five fungemia cases involved *C. tropicalis* (*n* = 2), *C. parapsilosis* (*n* = 2), and *C. famata* (*n* = 1), which closely mirrors the patterns observed in blood culture studies by Rahbar et al. [35], St-Germain et al. [36], and Sandven et al. [33].

In this study, the diagnostic utility of CHROMagar^TM^ *Candida* (CMA) was evaluated, revealing it to be a rapid, cost-effective method for the routine identification of clinically relevant yeasts. Our findings demonstrate that CMA can successfully identify *C. albicans* (light green), *C. tropicalis* (blue), and *C. krusei* (pink, rough colonies) with 100% sensitivity and specificity. These results are in excellent agreement with Nadeem et al. [37], who reported that CHROMagar^TM^ *Candida* easily differentiates *C. albicans*, *C. tropicalis*, and *C. krusei* with specificities and sensitivities of 99%, 98%, and 100%, respectively. In contrast, CMA could not differentiate between *C. parapsilosis* and *C. glabrata*, as both species produced identical pale pink colonies. Concurrently, microscopic examination of *Candida* growth on cornmeal agar supplemented with Tween 80 (CTA) successfully differentiated these species, with the notable exception of *C. famata* (*Debaryomyces hansenii*) isolates. This observation strongly supports the conclusions of Saeed et al. [38], who emphasized that identifying rare species remains challenging, noting that uncommon yeasts such as *C. guilliermondii*, *C. pelliculosa*, and *C. famata* (*Debaryomyces hansenii*) are frequently misidentified by direct inoculation on chromogenic media. Therefore, CMA and CTA can be implemented in tandem to verify key *Candida* species in a simple, rapid manner. Our findings concur with those of Koehler et al. [39], who observed that *C. parapsilosis*, *C. guilliermondii*, and *C. glabrata* yield overlapping pale pink colonies on chromogenic media but can be effectively resolved using CTA morphology.

However, two isolates phenotypically misidentified as *C. famata* (*Debaryomyces hansenii*) in our study were genotypically confirmed as *C. parapsilosis* via PCR and sequencing of the *ITS* region. This diagnostic discrepancy is concordant with the findings of Taverna et al. [16], who reported that out of six isolates phenotypically labeled as *C. famata*, four were genotypically resolved as *C. guilliermondii*. The primary significance of molecular identification lies in its unique capacity to overcome the limitations and misdiagnoses associated with conventional phenotypic methods. Methods using phenotypic media rely heavily on colony color variations and enzymatic profiles, which frequently overlap among rare non-*albicans Candida* species, leading to underdiagnosis of cryptic pathogens. These discrepancies are consistent with regional and international studies highlighting that relying solely on phenotypic traits underestimates non-*albicans* diversity in clinical settings. Thus, definitive molecular tools like PCR and *ITS* sequencing are indispensable for accurate epidemiological tracking and establishing species-specific antifungal regimens [18]. In our study, sequencing validated the phenotypic identification in all 45 *Candida* isolates, except for the two phenotypically misidentified *C. famata* strains that sequencing revealed to be *C. parapsilosis*. This misidentification can be explained by the high osmotolerance shared by *C. famata* and related species. Other species, particularly *C. guilliermondii* and *C. parapsilosis*, are frequently misidentified as *C. famata* based on phenotype alone. Because antifungal susceptibility profiles vary significantly across species, such diagnostic errors can profoundly influence a patient’s clinical course. A lack of clinical improvement should immediately alert clinicians to re-evaluate the diagnosis, implicated pathogens, or therapeutic choices [40]. These findings are strongly supported by studies conducted by Taverna et al. [16] and Mohammadnejad et al. [18].

Regarding antifungal susceptibility profiles, based on the disk diffusion and E-test methodologies, the 45 confirmed *Candida* isolates exhibited distinct susceptibility and resistance patterns. Specifically, fluconazole demonstrated excellent in vitro activity against *C. Albicans*, with 15/15 isolates classified as susceptible via disk diffusion, while the E-test revealed susceptibility in 10/15 isolates, an S-DD profile in 1/15, and resistance in 4/15. Conversely, fluconazole showed poor activity against non-*albicans* species, with high-level resistance and S-DD patterns as detailed in our findings, which parallel the observations reported by Tan et al. [41] and Hassan et al. [42]. Furthermore, regarding voriconazole, the E-test methodology across the total isolates revealed that 24/45 (53.33%) were susceptible, 8/45 (17.78%) were susceptible dose-dependent (S-DD), and 3/45 (6.67%) were confirmed as resistant, while *C. Albicans* showed potent activity with 15/15 (100%) susceptible via disk diffusion and 11/15 (73.33%) via the E-test. For other non-*albicans* species (excluding *C. famata*, which lacked established clinical breakpoints for azoles), voriconazole results aligned with broader trends noted by Magill et al. [43]. Additionally, regarding *C. glabrata* (*n* = 7), interpretation against voriconazole using the epidemiological cutoff value (ECV) of 0.5 mcg/mL revealed that 71.43% (5/7) exhibited MICs < 0.5 mcg/mL and were classified as wild-type (WT), while 28.57% (2/7) exhibited MICs > 0.5 mcg/mL and were categorized as non-wild-type (non-WT). These distinct variations underscore the critical need for routine susceptibility testing in clinical settings. The emergence of resistance or decreased susceptibility to traditional azoles like fluconazole among non-*albicans* isolates carries profound clinical implications, highlighting the danger of unguided therapies [44]. Consequently, these findings strongly support a clinical shift toward targeted, susceptibility-guided treatment protocols to improve patient outcomes and curb the further selection of resistant strains [45].

Real-time PCR assays were successfully deployed to screen for the presence of the *ALS*, *ALS3*, *HWP1*, and *SAP4* virulence genes among the isolates. The results revealed that *ALS1* was present in 86.7% of *C. albicans*, 42.8% of *C. glabrata*, and 22.2% of *C. parapsilosis* strains, whereas all *C. tropicalis* and *C. krusei* isolates were completely negative for this gene. The *HWP1* gene was detected in 93.33% of *C. albicans*, 57.14% of *C. glabrata*, and 28.6% of *C. tropicalis* strains. The *HWP1* surface protein is proposed as an essential substrate driving the covalent adherence of *C. albicans* to host epithelial cells [18]. Additionally, a comprehensive cooperative role for the *ALS1*, *ALS3*, and *HWP1* genes in biofilm formation has been hypothesized; numerous studies have demonstrated that the interaction between the *HWP1* surface protein and *ALS1*/*ALS3* adhesins is vital for the initiation and maturation of biofilms [18]. This variation in virulence gene profiles highlights a distinct comparative pattern: *C. albicans* isolates harbor a significantly higher burden of all tested pathogenicity factors than non-*albicans* species, which may contribute to the phenotypic virulence patterns observed in clinical settings. Interestingly, the *ALS3* gene was detected in more than 83% of isolates harboring the *HWP1* gene. Numerous studies have demonstrated that the *ALS3* gene of the *ALS* family and the *HWP1* gene play essential, complementary roles in *Candida* adherence and biofilm formation [46]. In this study, *C. albicans* and *C. glabrata* exhibited greater frequencies of both *HWP1* and *SAP4* genes compared to other species like *C. famata* and *C. krusei*. These data further support the hypothesis that the *HWP1* and *SAP4* genes play a major role in the initiation and progression of *Candida* infections across different anatomical tissue locations [19]. Additionally, these detection rates and virulence marker distributions are in accordance with recent Egyptian findings emphasizing the high pathogenic potential of clinical *Candida* isolates [Abdel-Aal et al., 2023] [47]. Furthermore, it should be noted that the detection of these virulence genes via PCR reflects gene presence rather than active transcriptional expression or functional protein production, which warrants future expression analyses.

### Study Limitations

Despite providing valuable clinical insights, several limitations of this study should be acknowledged. First, this investigation was designed as a single-center study conducted within a specific geographic area, which may restrict the immediate generalizability of the findings on a broader national scale. Second, the final sample size of 45 *Candida* isolates is relatively small, particularly for non-*albicans Candida*, which restricts generalized epidemiological assumptions. Additionally, susceptibility testing for caspofungin was not performed due to logistical constraints and because echinocandins are not yet routinely utilized as first-line empirical treatments in our specific clinical setting; instead, fluconazole and amphotericin B remain the primary therapeutic standards. Consequently, future multicenter, longitudinal studies incorporating larger patient cohorts, universal genomic sequencing, and a broader panel of contemporary antifungal agents are highly recommended to expand upon these findings.

## 5. Conclusions

- Although *Candida albicans* remains the leading cause of human candidiasis, non-*albicans Candida* species—such as *C. glabrata*, *C. tropicalis*, *C. krusei*, and *C. parapsilosis*—are increasingly emerging as opportunistic pathogens that exhibit enhanced resistance to conventional antifungal agents.
- Cornmeal agar supplemented with Tween 80 (CTA) represents a highly suitable and reliable phenotypic medium for the morphological differentiation and identification of distinct *Candida* species.
- Voriconazole demonstrated higher in vitro activity against the tested non-*albicans* isolates compared to fluconazole; however, clinical efficacy must be confirmed through prospective therapeutic trials.
- Phenotypic methods remain useful for routine initial screening of *Candida* species in resource-limited settings; however, molecular confirmation is essential for accurate identification of non-*albicans Candida* species and for characterization of strain-specific virulence factors.
- Nosocomial pathogenic bacteria such as *Staphylococcus aureus* frequently co-colonize with *C. albicans*, potentially forming polymicrobial biofilms that complicate the therapeutic process and worsen clinical outcomes in immunocompromised individuals.
- Further studies incorporating RT-qPCR are warranted to evaluate the transcript levels of these detected virulence genes under varying clinical and environmental conditions.

## Figures and Tables

**Table 1:** *ALS1*, *ALS3*, *HWP1*, and *SAP4* genes’ primer sequences.

Primer	Sequence
*ALS1*	
Forward:	ACCAGAAGAAACAGCAGGTG
Reverse:	GACTAGTGAACCAACAAATACCAG
*ALS3*	
Forward:	CCAAGTGTTCCAACAACTGAA
Reverse:	GAACCGGTTGTTGCTATGGT
*HWP1*	
Forward:	ATGACTCCAGCTGGTTC
Reverse:	TAGATCAAGAATGCAGC
*SAP4*	
Forward:	GAGTGTTCTTGCTTTCGCTTTA
Reverse:	TTGCCCACATCATTTCTACC

**Table 2:** Distribution of *45 Candida* species as detected by genotypic and phenotypic methods.

	*Candida albicans*	*Candida glabrata*	*Candida tropicalis*	*Candida krusei*	*Candida famata*	*Candida parapsilosis*	Not Defined
Sequencing of *ITS* as reference method	15	7	7	4	3	9	
CHROMagar (CMA)	15	0	7	4	5	0	14
Cornmeal agar with Tween 80 (CTA)	15	7	7	4	0	7	5

ITS: internal transcribed spacer region.

**Table 3:** Performance characteristics of CHROMagar^TM^ compared to sequencing of internal transcribed spacer regions (*ITS*) identification of isolated *Candida* species.

	Sequencing of ITS	CHROMagar (CMA)	Sensitivity	Specificity	PPV	NPV	Kappa Test
*C. albicans*	15	15	100%	100%	100%	100%	1.000
*C. glabrata*	7	Not identified	0%	100%	NA	84.44%	0.000
*C. tropicalis*	7	7	100%	100%	100%	100%	1.000
*C. krusei*	4	4	100%	100%	100%	100%	1.000
*C. famat* *a*	3	5	100%	95.24%	60%	100%	0.719
*C. parapsilosis*	9	7 not identified (2 misidentified as *C. famata*)	0%	100%	NA	80%	0.000

CHROMagar^TM^ Candida cannot reliably differentiate *C. parapsilosis* and *C. glabrata* due to overlapping colony colors, resulting in 0% diagnostic sensitivity on CHROMagar^TM^ alone. ITS: internal transcribed spacer; PPV: positive predictive value; NPV: negative predictive value; NA: not applicable.

**Table 4:** Performance characteristics of corn meal agar with Tween 80 compared to sequencing of internal transcribed spacer (*ITS*) region identification of isolated *Candida* species.

	Sequencing of ITS	Cornmeal Agar with Tween 80 (CTA)	Sensitivity	Specificity	PPV	NPV	Kappa Test
*C. albicans*	15	15	100%	100%	100%	100%	1.000
*C. glabrata*	7	7	100%	100%	100%	100%	1.000
*C. tropicalis*	7	7	100%	100%	100%	100%	1.000
*C. krusei*	4	4	100%	100%	100%	100%	1.000
*C. famat* *a*	3	Not identified	0%	100%	NA	93.33%	0.000
*C. parapsilosis*	9	7	77.8%	100%	100%	94.74%	0.837

ITS: internal transcribed spacer; PPV: positive predictive value; NPV: negative predictive value; NA: not applicable.

**Table 5:** Antifungal susceptibility patterns (*N* = 45) of *Candida* species, identified using the disk diffusion method.

*Candida* Species	Antifungal Agent	Antifungal Susceptibility			
		S (%)	CI (95%)	R (%)	CI (95%)
*C. albicans* (No. = 15)	Fluconazole	15 (100%)	(78.2–100%)	0 (0%)	0–21.8%
	Voriconazole	15 (100%)	(78.2–100%)	0 (0%)	0–21.8%
	Amphotericin B	15 (100%)	(78.2–100%)	0 (0%)	0–21.8%
	Micafungin	15 (100%)	(78.2–100%)	0 (0%)	0–21.8%
*C. glabrata* (No. = 7)	Fluconazole	5 (71.42%)	29–96.3%	2 (28.6%)	3.7–71%
	Voriconazole	6 (85.71%)	42.1–99.6%	1 (14.3%)	0.4–57.9%
	Amphotericin B	6 (85.71%)	42.1–99.6%	1 (14.3%)	0.4–57.9%
	Micafungin	7 (100%)	59–100%	0 (0%)	0–41%
*C. tropicalis* (No. = 7)	Fluconazole	4 (57.14%)	18.4–99.6%	3 (42.8%)	9.9–81.6%
	Voriconazole	6 (85.71%)	42.1–99.6%	1 (14.29%)	0.45–57.9%
	Amphotericin B	7 (100%)	59–100%	0 (0%)	0–41%
	Micafungin	7 (100%)	59–100%	0 (0%)	0–41%
*C. krusei* (No. = 4)	Fluconazole	0 (0%)	0–60%	4 (100%)	39.8–100%
	Voriconazole	4 (100%)	39.8–100%	0 (0%)	0–60.2%
	Amphotericin B	4 (100%)	39.8–100%	0 (0%)	0–60.2%
	Micafungin	4 (100%)	39.8–100%	0 (0%)	0–60.2%
*C. parapsilosis* (No. = 9)	Fluconazole	6 (66.66%)	29.9–92.5%	3 (33.3%)	7.5–70.1%
	Voriconazole	9 (100%)	66.4–100%	0 (0%)	0–33.6%
	Amphotericin B	9 (100%)	66.4–100%	0 (0%)	0–33.6%
	Micafungin	8 (88.9%)	51.8–99.7%	1 (11.11%)	0.3–48.2%
*C. famata* (No. = 3)	Fluconazole	0 (0%)	0–70.8%	3 (100%)	29.2–100%
	Voriconazole	0 (0%)	0–70.8%	3 (100%)	29.2–100%
	Amphotericin B	3 (100%)	29.2–100%	0 (0%)	0–70.8%
	Micafungin	1 (33.33%)	0.8–90.6%	2 (66.665)	9.4–99.2%

N.B: S (susceptible), R (resistant), and CI (95% Confidence Interval).

**Table 6:** Fluconazole’s in vitro activity against different isolated *Candida* species as assessed via the E-test.

*Candida* Species	Sensitive			S-DD			Resistant		
	MIC (µg/mL)	No (%)	CI 95%	MIC (µg/mL)	No	CI 95%	MIC (µg/mL)	No	CI 95%
*C. albicans* (*n* = 15)	≤2	10 (66.67%)	38.4–88.2%	4	1 (6.67%)	0.2–31.9%	≥8	4 (26.67%)	7.8–55.1%
*C. glabrata* (*n* = 7)	-	0 (0%)	0–41.0	≤32	5 (71.43%)	29–96.3%	>64	2 (28.57%)	3.75–71%
*C. tropicalis* (*n* = 7)	≤2	2 (28.57%)	3.7–71%	4	1 (14.29%)	0.45–57.9%	≥8	4 (57.14%)	18.4–90.1%
*C. krusei* (*n* = 4)	-	0 (0%)	0–60.2%	-	0 (0%)	0–60.2%	•	4 (100%)	39.85–100%
*C. parapsilosis* (*n* = 9)	≤2	3 (33.33%)	7.5–70.1%	4	1 (11.11%)	0.35–48.2%	≥8	5 (55.56%)	21.2–86.3%
*C. famata* (*n* = 3)	≤2	0 (0%)	0–70.8%	4	0 (0%)	0–70.8%	≥8	3 (100%)	29.2–100%
Total (*n* = 45)		15 (33.33%)	205–49%		8 (17.78%)	8–32.1%		22 (48.89%)	33.7–64.2%

S-DD: Susceptible-dose dependent. • intrinsically resistant.

**Table 7:** Voriconazole’s in vitro activity against different isolated *Candida* species as assessed via the E-test.

*Candida* Species	Sensitive				S-DD		Resistant		
	MIC	No (%)	CI 95%	MIC	No (%)	CI 95%	MIC	No (%)	CI 95%
*C. albicans* (*n* = 15)	≤0.125	11 (73.33%)	44.9–91.3%	0.25–0.5	4 (26.67%)	7.8–55.1%	≥1	0 (0%)	0–21.8%
*C. glabrata* (*n* = 7)	-	-	-	-		-	-	-	-
*C. tropicalis* (*n* = 7)	≤0.125	4 (57.14%)	18.4–90.1%	0.25–0.5	2 (28.57%)	0.4–57.9%	≥1	1 (14.3%)	0.4–57.9%
*C. krusei* (*n* = 4)	≤0.5	3 (75%)	19.4–99.4%	1	0 (0%)	0–60.2%	≥2	1 (25%)	0.65–80.6%
*C. parapsilosis* (*n* = 9)	≤0.125	6 (66.67%)	29.9–92.5%	0.25–0.5	2 (22.22%)	2.8–60%	≥1	1 (11.11%)	0.35–48.2%
*C. famata* (*n* = 3)	-	-	-	-	-	-	-	-	-
Total (*n* = 45)		24 (53.33%)	38.7–67.5%		8 (17.78%)	8–32.1%		3 (6.67%)	1.8–18.1%

N.B: For *Candida glabrata* and *Candida famata*, clinical susceptibility categories (Sensitive, S-DD, Resistant) are not applicable (-) due to the lack of established CLSI clinical breakpoints. *C. glabrata* isolates were evaluated using Epidemiological Cutoff Values (ECVs) instead.

**Table 8:** The prevalence of *ALS1*, *ALS3*, *HWP1* and *SAP4* genes in different *Candida* species.

*Candida* Species (No. 45)	*ALS1*	CI 95%	*ALS3*	CI 95%	*HWP1*	CI 95%	*SAP4*	CI 95%
*C. albicans* (No. 15)	13 (86.7%)	59.5–98.3%	13 (86.7%)	59.5–98.3%	14 (93.33%)	68.15–99.8%	11 (73.33%)	44.95–92.2%
*C. glabrata* (No. 7)	3 (42.8%)	9.9–81.6%	5 (71.43%)	29–96.3%	4 (57.14%)	18.4–90.1%	4 (57.14%)	18.4–90.1%
*C. tropicalis* (No. 7)	0	0–41%	1 (14.3%)	0.4–57.9%	2 (28.6%)	3.7–71%	0	0–41%
*C. krusei* (No. 4)	0	0–60.2%	0	0–60.2%	0	0–60.2%	1 (25%)	0.6–80.6%
*C. parapsilosis* (No. 9)	2 (22.2%)	2.8–60%	0	0–33.6%	0	0–33.6%	0	0–33.6%
*C. famata* (No. 3)	0	0–70.8%	0	0–70.8%	0	0–70.8%	1(33.33%)	0.8–90.6%
Total frequency in all isolated species (NO 45)	18 (40%)	25.7–55.7%	19 (42.22%)	27.7–57.9%	20 (44.44%)	29.8–60%	17 (37.78%)	23.8–53.5%

Note: Statistical analysis test was performed via Fisher’s exact to compare *C. albicans* against total non-*albicans Candida* species. The prevalence of *ALS1*, *ALS3*, *HWP1*, and *SAP4* genes was significantly higher in *C. albicans* compared to non-*albicans* species (*p* < 0.001 for *ALS1*, *ALS3*, and *HWP1*; *p* = 0.001 for SAP4).

## Data Availability

The data presented in this study are available on request from the corresponding author due to privacy.

## Supplementary Materials

The following supporting information can be downloaded at https://www.mdpi.com/article/10.3390/pathogens15080878/s1, Table S1: Distribution of the isolated organisms among the 211 clinical specimens collected from patients. Table S2: Association of *Candida* infections and *Candidemia* with the clinical profile of patients (200 patients).

## Abbreviations

The following abbreviations are used in this manuscript:

ICUs	intensive care units
ITS	internal transcribed spacer
NAC	non-albicans Candida;
CLSI	Clinical and Laboratory Standards Institute
ALS	Agglutinin-Like Sequence family
HWP1	Hyphal Wall Protein 1
SAP	Secreted Aspartyl Proteinase
rDNA	ribosomal DNA
ESRD	end-stage renal disease
NICU	neonatal ICU
CSF	cerebrospinal fluid
BAL	bronchoalveolar lavage
SDA	Sabouraud Dextrose Agar
UTIs	urinary tract infections
MICs	Minimum Inhibitory Concentrations
