# Supplementary material for: Phenotypic and Molecular Assessment of Candida Species and Real-Time PCR Detection of Selected Virulence Genes in Immunocompromised Patients Admitted to Intensive Care Units
Source: Pathogens. 2026 Aug 21;15(8):878. doi: 10.3390/pathogens15080878 (PMC13516977; doi:10.3390/pathogens15080878)
Supplement: Supplementary file 1 [file pathogens-15-00878-s001.zip › pathogens-4463991-supplementary.pdf]

Table S1: Distribution of the isolated organisms among the 211 clinical specimens collected from patients.

Source of specimens	No. of specimens	Pure *Candida* isolates		*Aspergillus* isolates		Mixed *Candida* & bacteria		Bacteria isolates		No growth	
		No.	%	No.	%	No.	%	No.	%	No	%
**Blood culture**	121 57.35%	8	6.6%	6	5%	6	5 %	58	47.9%	43	35.5%
**Urine& Foley catheter**	44 20.8%	22	50%	0	0%	3	6.8%	18	40.9%	1	2.27%
**CSF**	6 2.85%	0	0%	0	0%	0	0%	3	50%	3	50%
**Sputum& bronchoalveolar lavage**	40 19%	3	7.5%	5	12.5%	3	7.5%	28	70%	1	2.5%
**Total No. of specimens**	211 100%	33	15.6%	11	5.2%	12	5.7%	107	50.7%	48	22.7%
**Total No. of positive- culture specimens (163)**		33	20.2%	11	6.7%	12	7.3%	107	65.6%	-	-
**Total No. of Candida isolates (45)**		33	73.3%			12	26.7%				
**Total No. of bacterial isolates (119)**						12	10%	107	90%		

Table S2: Association of *Candida* infections and *Candidemia* with the clinical profile of patients (200 patients)

Predisposing factors	*Candida* infections		OR (CI 95%)	P- value	*Candidaemia*		OR (CI 95%)	P- value
	Positive (No. =34)	Negative (No. = 166)			Positive (No. = 11)	Negative (No. = 189)		
**Antibiotic treatment Yes (No. = 47)**<br><br>**No (No. = 153)**	34<br><br>0	13<br><br>153	802.2 (46.8 – 3745.0)<br><br>Reference	<0.0001	10<br>1	34<br>155	45.59 (5.63 – 368.8)<br><br>Reference	< 0.0001
**DM**<br>**Yes (No. = 38)**<br>**No (No. = 162)**	20<br>14	18<br>148	11.75 (5.14 – 26.83)<br>Reference	<0.0001	2<br>9	36<br>153	0.94 (0.19 – 4.59)<br>Reference	0.943
**Burn**<br>**Yes (No. = 25)**<br>**No (No. = 175)**	5<br>29	20<br>146	1.26(0.44 – 3.59)<br>Reference	0.669	1<br>10	24<br>165	0.69 (0.08 – 5.64)<br>Reference	0.725
**Neonatal period**<br>**Yes (No. = 21)**<br>**No (No. = 179)**	13<br>21	8<br>158	12.23(4.55-32.86)<br>Reference	< 0.0001	5<br>6	16<br>173	9.01 (2.46 – 33.00) Reference	<0.0001
**Renal failure**<br>**Yes (No. = 30)**<br>**No (No. = 170)**	4<br>30	26<br>140	0.72 (0.16 – 3.44)<br>Reference	0.561	2<br>9	28<br>161	1.28 (0.26 – 6.27)<br>Reference	0.761
**Liver failure**<br>**Yes (No. = 15)**<br>**No (No. = 185)**	2<br>32	13<br>153	0.74 (0.16 – 3.44)<br>Reference	0.694	1<br>10	14<br>175	1.25 (0.15 – 10.36) Reference	0.836
**Malignancy**<br>**Yes (No. = 5)**<br>**No (No. = 195)**	1<br>33	4<br>162	2.63 (0.73 – 9.47)<br>Reference	0.856	1<br>10	4<br>185	4.63 (0.48 – 44.57) Reference	0.149
**Parenteral nutrition**<br>**Yes (No. = 12)**<br>**No (No. = 188)**	4<br>30	8<br>158	3.65 (1.31 – 10.21)<br>Reference	0.12	4<br>7	8<br>181	12.93 (3.09 – 54.12)<br>Reference	<0.0001
**Urinary catheter**<br>**Yes (No. = 18)**<br>**No (No. = 182)**	7<br>27	11<br>155	3.65 (1.31– 10.21)<br>Reference	0.009	2<br>9	16<br>173	2.40 (0.47 – 12.30) Reference	0.273
**Surgery**<br>**Yes (No. = 5)**<br>**No (No. = 195)**	1<br>33	4<br>162	14.14 (2.61 – 76.58)<br>Reference	0.856	1<br>10	4<br>185	4.63 (0.48 – 44.57) Reference	0.149
**Central venous line**<br>**Yes (No. = 7)**<br>**No (No. = 193)**	5<br>29	2<br>164	14.14(2.61-76.58)<br>Reference	<0.001	4<br>7	3<br>186	35.43 (7.07 – 177.58)<br>Reference	<0.0001

N.B., More than one predisposing factor may be present in the same patient.

P-values were calculated using Pearson's Chi-square test, or Fisher's exact test when expected cell counts were less than 5. Odds Ratios (OR) and 95% Confidence Intervals (CI) were determined to evaluate the strength of association."
